# Efficacy of traditional Chinese medicine nursing for lung cancer: a systematic review and meta-analysis using the Donabedian model to investigate heterogeneity

**DOI:** 10.3389/fpubh.2026.1833561

**Published:** 2026-08-26

**Authors:** Ting Duan, Yuxin Liu

**Affiliations:** 1 Affiliated Hospital of Jinggangshan University, Ji'an, China; 2 School of Nursing, Jinggangshan University, Ji'an, China

**Keywords:** Donabedian model, heterogeneity, lung cancer, Meta-analysis, psychological outcomes, quality of life, traditional Chinese medicine nursing

## Abstract

**Background:**

Lung cancer patients frequently experience a reduced quality of life and psychological distress during treatment. Traditional Chinese Medicine (TCM) nursing shows promise, but substantial heterogeneity in the evidence base limits clinical interpretation.

**Methods:**

A comprehensive literature search identified 15 randomized controlled trials (RCTs) involving 1,135 participants. A meta-analysis was conducted using RevMan 5.4 and Stata 17. Following the Donabedian framework, data were categorized into structure, process, and outcome dimensions to explore sources of heterogeneity.

**Results:**

In the primary analysis, TCM nursing significantly improved all outcomes. For the Karnofsky Performance Status (KPS) score, the primary random-effects analysis showed a significant improvement (MD = 7.18, 95%CI 4.29–10.08). The sensitivity analysis excluding one outlier yielded a consistent effect (MD = 8.71, 95%CI 6.49–10.92). For the Self-Rating Depression Scale (SDS) score, the primary analysis showed a significant reduction (MD = −5.94, 95%CI −6.96 to −4.92). The sensitivity analysis excluding one outlier confirmed the robustness of these results (MD = −5.74, 95%CI −6.39 to −5.09). For the remaining outcomes with low heterogeneity, primary fixed-effects analysis revealed that TCM nursing significantly reduced Self-Rating Anxiety Scale (SAS) scores (MD = −4.65, 95%CI −5.28 to −4.03) and Visual Analog Scale (VAS) pain scores (MD = −1.46, 95%CI −1.56 to −1.36). Additionally, TCM nursing lowered the incidence of gastrointestinal reactions (RR = 0.19, 95%CI 0.06–0.58) and improved nursing compliance (RR = 1.24, 95%CI 1.10–1.39) (all *p* < 0.01). The subgroup analysis confirmed that comprehensive TCM care was more effective than emotion regulation alone for treating depression (*p* = 0.01). Intervention timing was suggested as a potential hypothesis-generating source of heterogeneity via sensitivity analysis. Structural factors (staff training, equipment, and qualifications) were critically underreported across all studies included in the analysis.

**Conclusion:**

TCM nursing significantly improves multidimensional outcomes for lung cancer patients. The Donabedian model effectively identifies sources of process-related heterogeneity and highlights critical structural reporting gaps that require attention in future research.

**Systematic review registered:**

PROSPERO, ID https://www.crd.york.ac.uk/PROSPERO/view/CRD420261339100.

## 1 Introduction

Lung cancer is the leading cause of cancer-related mortality worldwide, representing a substantial disease burden as it ranks first globally in both incidence and mortality rates (1–4). Conventional mainstream therapies can effectively control the progression of lung cancer, but they are often accompanied by a variety of adverse reactions, which seriously affect patients’ quality of life (5). TCM nursing, which emphasizes a holistic approach and syndrome-differentiated care (6), demonstrates unique potential in alleviating symptoms and mitigating chemotherapy toxicity (7–9); however, its clinical application for lung cancer faces significant challenges (10). Practices vary widely across institutions, protocols lack standardization, and systematic quality evaluation frameworks remain underdeveloped. More fundamentally, the evidence base for TCM nursing, while generally positive, is characterized by substantial heterogeneity that limits confident interpretation and clinical translation (11).

The Three-Dimensional Quality Structure Theory (Structure-Process-Outcome) proposed by Donabedian is a classic theoretical framework for evaluating the quality of medical and nursing services, providing a systematic perspective for the comprehensive evaluation of nursing efficacy (12–14). At present, the application of this theory in the field of TCM nursing for lung cancer remains in the exploratory stage. Applying this model to the current evidence base for TCM nursing in lung cancer offers a systematic approach to investigating heterogeneity and provides a theoretical basis for standardizing TCM nursing practice and optimizing research design.

Therefore, this study has three specific objectives. First, it aims to systematically evaluate the efficacy of TCM in nursing lung cancer patients through meta-analysis of RCTs. Second, it will utilize the Donabedian Structure-Process-Outcome model as an analytical framework to systematically investigate the sources of heterogeneity in the existing evidence base. Third, the study seeks to identify the specific structural and process factors that may moderate the therapeutic effects of TCM nursing, thereby providing insights to standardize future research and optimize the clinical practice of TCM nursing for lung cancer.

## 2 Materials and methods

This systematic review and meta-analysis was conducted in strict accordance with the PRISMA 2020 guidelines (15), and the research protocol was registered in PROSPERO with registration number:2026 CRD420261339100 (16).

### 2.1 Search strategy

The electronic databases included English databases, such as PubMed, Web of Science, Embase, and Cochrane Library, and Chinese databases, such as CNKI, VIP, and WanFang. The search period extended from the establishment of each database to 1 August 2025. The search strategy combined Medical Subject Headings (MeSH) terms and free words, with keywords including “lung cancer,” “traditional Chinese medicine nursing,” “syndrome differentiation nursing,” “three-dimensional quality structure,” “structure-process-outcome,” and “randomized controlled trial.” The detailed search strategy for each database is provided in the Supplementary material (Appendix 1). The original search was performed on 1 August 2025. Prior to final submission, the searches were re-run on 24 July 2026 to verify the relevancy of the findings; no additional studies that met the inclusion criteria were identified, and the final set of 15 RCTs remained unchanged.

### 2.2 Inclusion and exclusion criteria

The inclusion criteria are as follows: (1) study type: randomized controlled trials (RCTs), regardless of language and blinding method; (2) study subjects: patients with pathologically confirmed lung cancer, regardless of disease stage, gender, and age; (3) intervention measures: the control group received routine clinical nursing, and the experimental group received TCM nursing on the basis of routine nursing (This could include either a single TCM nursing measure or a comprehensive TCM nursing measure); (4) outcome indicators: at least one outcome measure related to the three dimensions of structure, process, and outcome was reported, including the KPS score, the SAS score, the SDS score, the VAS pain score, incidence of gastrointestinal reactions, and nursing compliance.

The exclusion criteria are as follows: (1) non-RCT studies such as cohort studies, case–control studies, cross-sectional studies, reviews, case reports, and conference abstracts; (2) studies on animal experiments or basic research; (3) studies with inconsistent research subjects (non-lung cancer patients); (4) studies with incomplete data, unextractable outcome indicators, or duplicate publications; and (5) studies with unreasonable study design or obvious methodological defects.

### 2.3 Literature screening and data extraction

Two researchers independently screened the literature according to pre-set inclusion and exclusion criteria. First, the titles and abstracts were read for preliminary screening to exclude irrelevant literature; then the full texts of the remaining literature were read for re-screening to determine finally included studies. In case of disagreement between the two researchers, a third researcher was invited to facilitate discussion and reach consensus.

A unified data extraction table was formulated to extract the relevant data of the included studies independently by two researchers, including: (1) basic information of the study: author, publication year, country, and study design; (2) research subjects: sample size, baseline characteristics of patients (disease stage, age, gender); (3) intervention measures: TCM nursing measures in the experimental group, routine nursing measures in the control group, intervention course; (4) outcome measures: type of outcome measure, measurement data, count data and corresponding statistical results; and (5) methodological quality items: random sequence generation, allocation concealment, and blinding method. Cross-verification was conducted on the extracted data, and missing data were obtained from the original research author as far as possible for supplementation.

### 2.4 Quality assessment of studies

Two researchers independently evaluated the methodological quality of the included RCTs using the Cochrane Risk of Bias Tool (17), which mainly evaluates the risk of bias from seven domains: random sequence generation and allocation concealment (selection bias), blinding of participants and researchers (performance bias), blinding of outcome assessors (detection bias), incomplete outcome data (attrition bias), and selective reporting (reporting bias), and other bias. Each dimension was rated as “low risk of bias,” “unclear risk of bias,” or “high risk of bias.” Cross-verification was conducted on the evaluation results, and any disagreements were resolved by a third researcher.

### 2.5 Data synthesis and analysis

RevMan 5.4 and Stata 17 software were used for statistical analysis. Measurement data were expressed as mean difference (MD) and 95% confidence interval (CI), and count data were expressed as risk ratio (RR) and 95%CI. Pre-specified statistical analysis strategies: (1) Heterogeneity test: *χ*^2^ test and *I*^2^ statistic were used to evaluate the statistical heterogeneity between studies. If *p* ≥ 0.1 and *I*^2^ ≤ 50%, it indicated low heterogeneity, and the fixed-effect model was used; if *p* < 0.1 and *I*^2^ > 50%, it indicated high heterogeneity, and the random-effects model was used after exploring the sources of heterogeneity (18); (2) Outlier handling: For outcomes with high heterogeneity, single study omission was used to identify outlier studies, and the reasons for outliers were analyzed from clinical and methodological perspectives before re-analysis; (3) Subgroup analysis: Conducted according to intervention type (emotion regulation intervention, comprehensive TCM nursing intervention) to explore the sources of heterogeneity; (4) Sensitivity analysis: Performed by sequentially omitting a single study and excluding high-risk bias studies to evaluate the robustness of the results; (5) Publication bias: Evaluated by funnel plot, Egger’s test and Begg’s test. If the funnel plot was basically symmetric and Egger’s and Begg’s tests had *p* > 0.05, this indicated no significant publication bias. The test level was *α* = 0.05, and a *p*-value <0.05 was considered statistically significant.

## 3 Results

### 3.1 Literature search results

**Figure 1:**
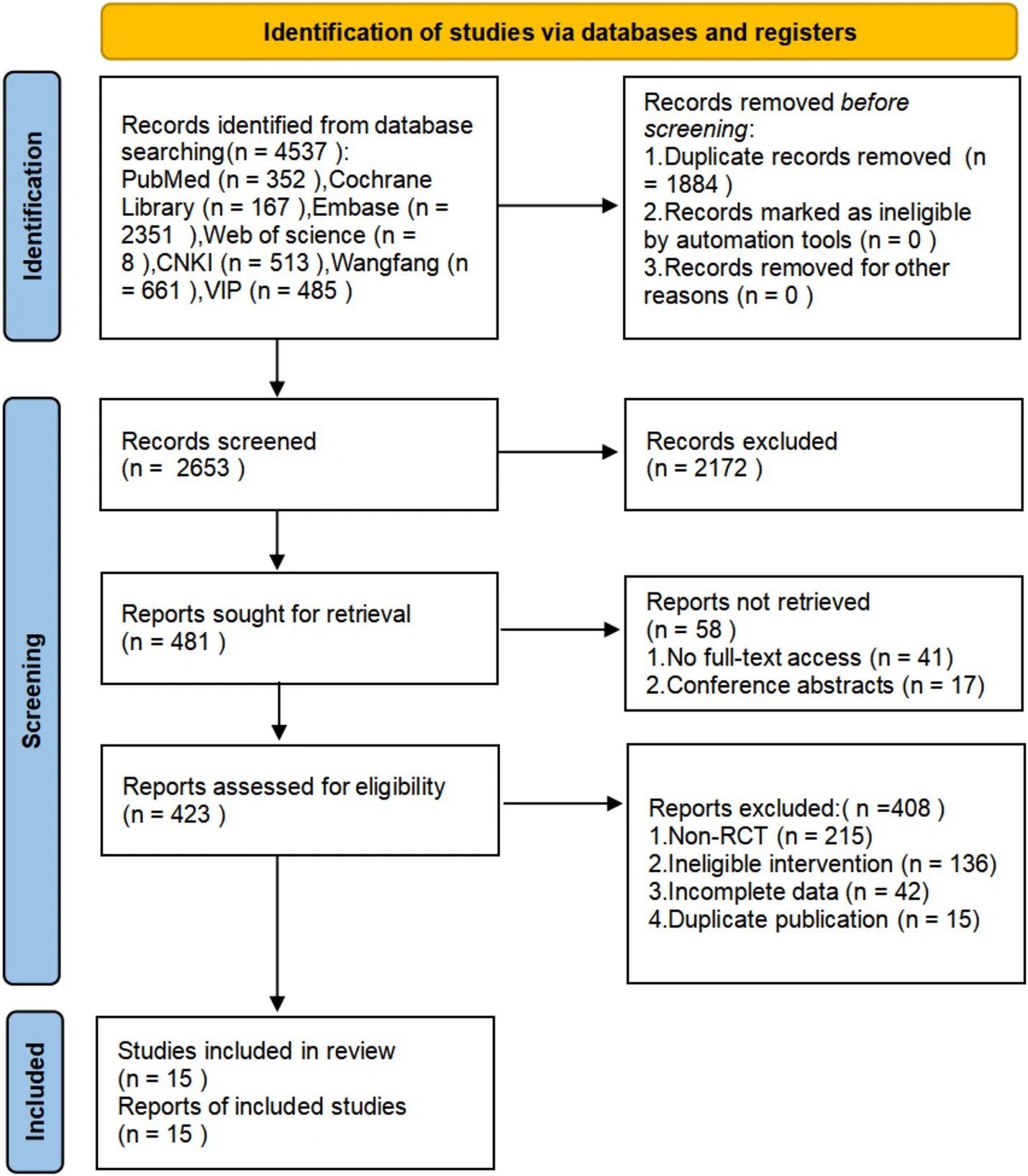
PRISMA flow diagram.

A total of 4,537 relevant studies were initially retrieved from various databases. After removing 1884 duplicate literatures using EndNote 21 software, 2,653 literatures were left for title and abstract screening, and 2,172 irrelevant literatures were excluded. The remaining 481 literatures were retrieved for full-text reading; 58 literatures could not be obtained, and 423 literatures were finally included in the eligibility evaluation. According to the inclusion and exclusion criteria, 408 literatures were excluded (215 non-RCTs, 136 ineligible intervention measures, 42 incomplete data, 15 duplicate publications). Finally, 15 RCTs were included in the meta-analysis, with a total of 1,135 participants, including 568 in the experimental group and 567 in the control group. The PRISMA flow chart for the literature screening is shown in Figure 1.

### 3.2 Basic characteristics of included studies

The 15 (19–33) included studies were published from 2019 to 2024, including 7 Chinese literatures (22, 24, 26–28, 30, 33) and 8 English literatures (19–21, 23, 25, 29, 31, 32). The sample size of each study ranged from 38 to 100 cases. The TCM nursing measures adopted in the experimental group include five-element music nursing, emotional nursing, acupoint application, comprehensive TCM nursing, and integrated traditional Chinese and Western medicine nursing. The outcome measures mainly included KPS score, SAS score, SDS score, VAS score, incidence of gastrointestinal reactions, and nursing compliance. The intervention duration ranged from 5 to 90 days across studies; however, the duration was reported in only 5 of the 15 included studies. Regarding intervention timing, five studies (19, 25, 30–32) implemented TCM nursing during chemotherapy, two studies (20, 24) postoperatively, one study (22) after chemotherapy, and the remaining seven studies did not report this information. The incomplete reporting of both duration and timing precluded formal subgroup analysis or meta-regression for these process factors. Detailed characteristics of each study, including duration and timing, are presented in Table 1.

**Table 1:** Characteristics of the included studies.

No.	Author, year	Sample (I/C)	Sex (M/F) (I/C)	Intervention	Control	Duration (days)	Timing	Outcomes
1	Zhao et al., 2024 (21)	40/40	Not reported	Five-element music and positive psychology	Routine care	5–7 days	Not reported	②③
2	Zhou 2024 (20)	40/40	(31/9)/(30/10)	Five-element music and TCM emotional nursing	Routine care	Not reported	Post-operative	②⑤
3	Yang 2024 (23)	39/39	(22/17)/(20/19)	Routine care and acupoint application	Routine care	28 days	Not reported	③⑤
4	Wang et al. 2023 (25)	37/37	(22/15)/(21/16)	TCM nursing	Routine care	Not reported	During chemotherapy	②④
5	Wu 2022 (24)	31/31	Not reported	TCM nursing	Routine care	Not reported	Post-operative	②
6	Mei and Li 2022 (27)	19/19	(12/7)/(13/6)	Routine care and TCM emotional nursing	Routine care	Not reported	Not reported	①②⑤
7	Feng 2022 (31)	40/40	(27/13)/(25/15)	TCM nursing	Routine care	Not reported	During chemotherapy	②④
8	Dong and Guan 2022 (32)	36/36	(25/11)/(27/9)	Routine care and TCM nursing	Routine care	13 days	During chemotherapy	②
9	Yin et al. 2021 (22)	45/45	(27/18)/(26/19)	Routine care and TCM nursing	Routine care	Not reported	Post-chemotherapy	②④
10	Kang and Wang 2020 (29)	50/50	(39/11)/(37/13)	TCM nursing	Routine care	Not reported	Not reported	②
11	Gong 2020 (30)	22/22	(11/11)/(10/12)	Comprehensive TCM nursing	Routine care	Not reported	During chemotherapy	①
12	Qi 2020 (26)	32/32	(18/14)/(15/17)	Routine care and TCM nursing	Routine care	Not reported	Not reported	②④
13	Li 2019 (28)	46/46	(25/21)/(26/20)	Routine care and acupoint application	Routine care	28 days	Not reported	③⑤
14	Zhu et al. 2019 (19)	47/47	(22/25)/(21/26)	Integrated Traditional Chinese and Western Medicine nursing	Routine care	Not reported	During chemotherapy	④⑤
15	Bao et al. 2019 (33)	44/43	Not reported	Routine care and Comprehensive TCM nursing	Routine care	90 days	Not reported	②

No., number; I/C, intervention/control; M/F, male/female. ① Nursing compliance; ② Self-Rating Anxiety Scale (SAS) and Self-Rating Depression Scale (SDS) scores; ③ Karnofsky Performance Status (KPS) score; ④ incidence of gastrointestinal reactions; and ⑤ visual analog scale (VAS) pain score.

### 3.3 Risk of Bias assessment results

**Figure 2:**
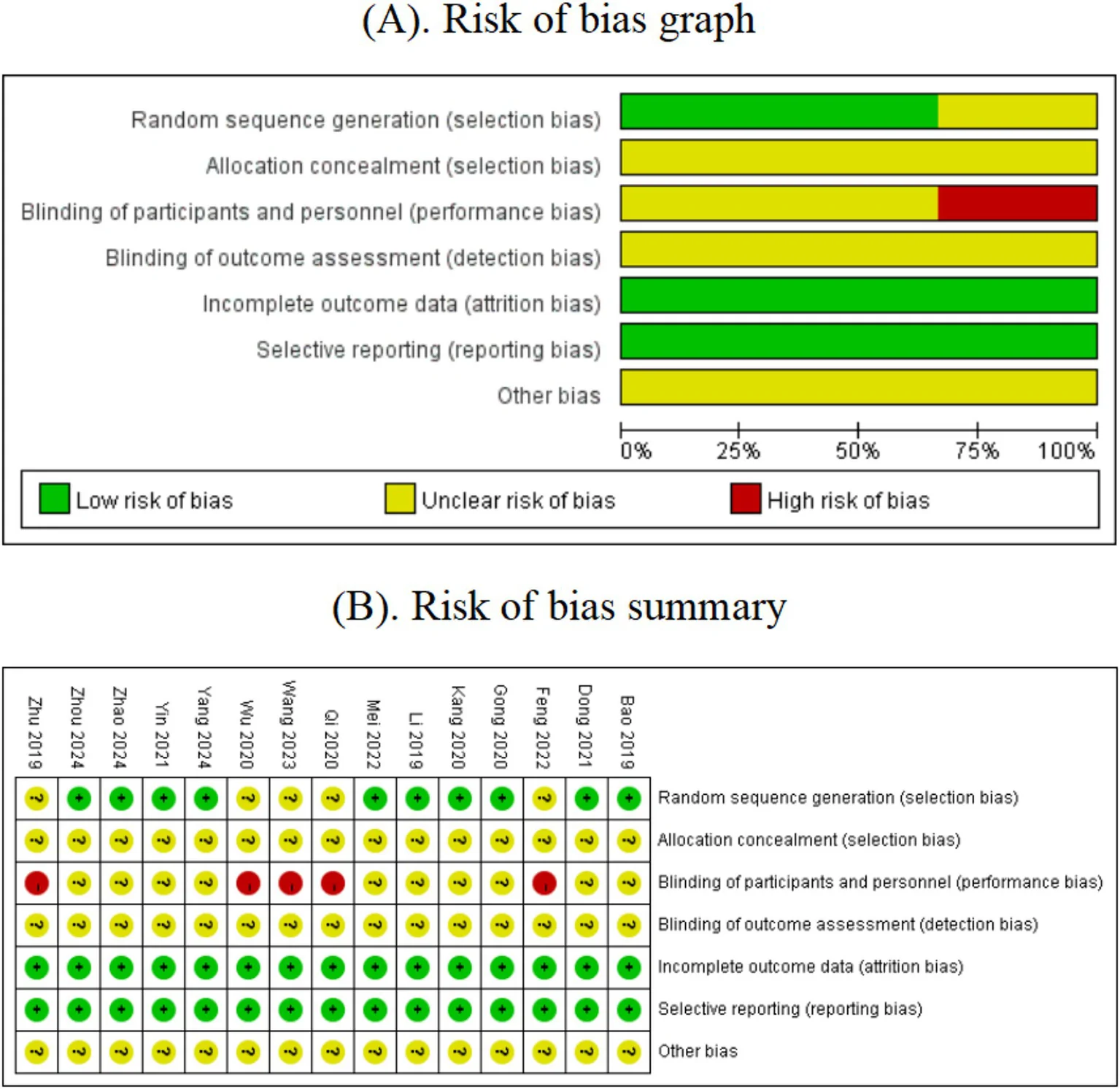
Risk of bias assessment using the Cochrane Risk of Bias Tool. **(A)** Risk of bias graph; **(B)** Risk of bias summary.

The results of the Cochrane Risk of Bias Tool evaluation showed that: eight studies (21–23, 27–30, 33) adopted a random number table for random sequence generation (low risk), two studies (20, 32) only stated random allocation without specifying the method (unclear risk), and five studies (19, 24–26, 31) did not report the random method (unclear risk); all studies did not report the allocation concealment method (unclear risk); five studies (19, 24–26, 31) explicitly stated that no blinding was used (high risk); all studies had complete outcome data and no selective reporting (low risk); no other obvious sources of bias were found in all studies (low risk). The risk of bias graph and summary chart are shown in Figure 2.

### 3.4 Meta-analysis results

#### 3.4.1 Process quality: nursing compliance

**Figure 3:**
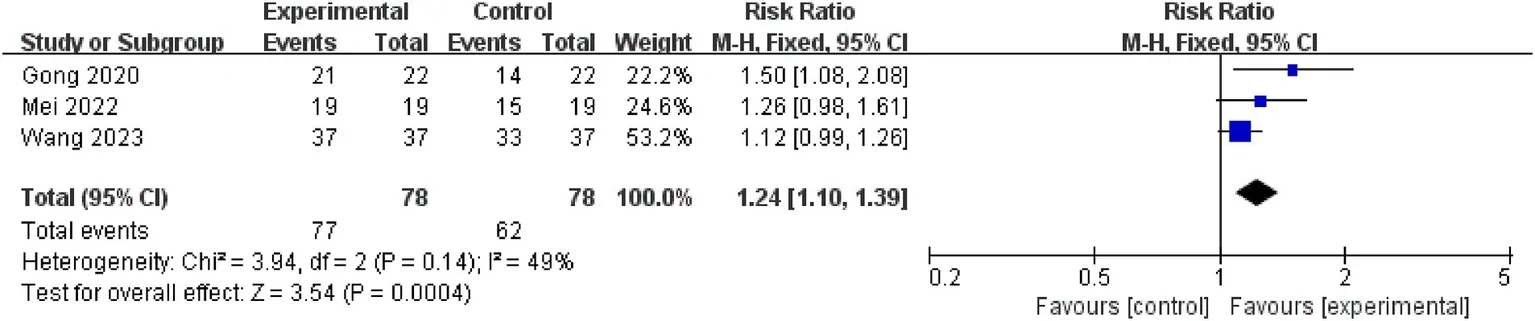
Forest plot of nursing compliance.

Three studies (25, 27, 30) reported the nursing compliance of lung cancer patients. The heterogeneity test showed low heterogeneity among studies (*p* = 0.14, *I*^2^ = 49%), and the fixed-effect model was used for analysis. The results showed that the nursing compliance rate of the experimental group receiving TCM nursing was significantly higher than that of the control group (RR = 1.24, 95%CI 1.10–1.39, *p* = 0.0004) (Figure 3).

#### 3.4.2 Outcome quality

##### 3.4.2.1 Anxiety and depression (SAS and SDS scores)

Ten studies (20–22, 24, 26, 27, 29, 31–33) reported SAS and SDS scores for evaluating anxiety and depression in patients.

**Figure 4:**
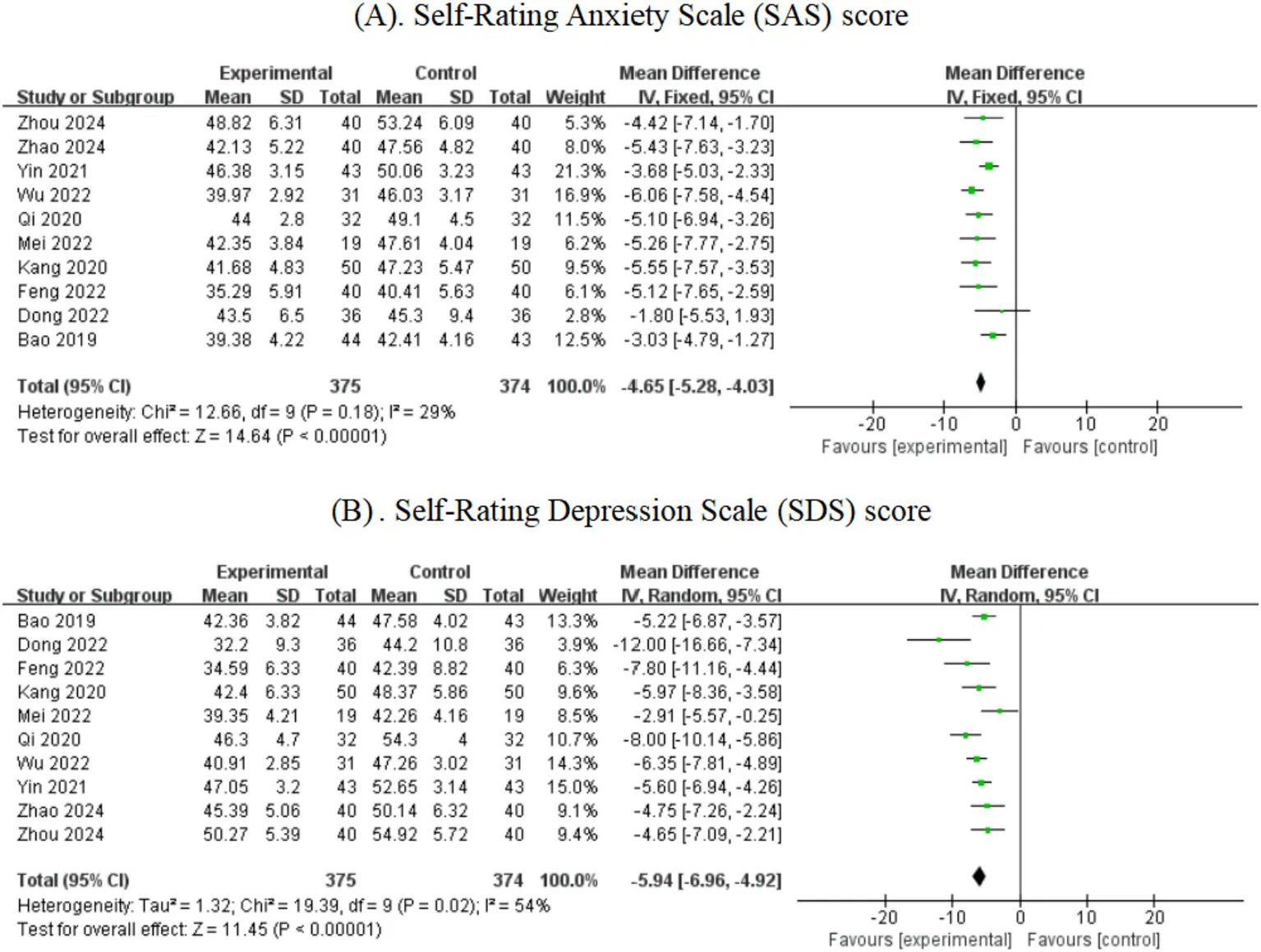
Forest plots of anxiety and depression outcomes. **(A)** Self-Rating Anxiety Scale (SAS) score (primary analysis including all 10 studies); **(B)** Self-Rating Depression Scale (SDS) score (full-sample primary analysis including all 10 studies, random-effects model, with sensitivity analysis after excluding Dong et al. discussed in the text).

For the SAS score (primary analysis, all 10 studies), the heterogeneity test showed low heterogeneity (*p* = 0.18, *I*^2^ = 29%), and the fixed-effects model was used for analysis. The results showed that the SAS score of the experimental group was significantly lower than that of the control group (MD = −4.65, 95%CI −5.28 to −4.03, *p* < 0.00001) (Figure 4A).

For SDS score, we first conducted a full-sample primary analysis including all 10 studies. The initial heterogeneity test showed moderate-to-high heterogeneity (*p* = 0.02, *I*^2^ = 54%), and the random-effects model showed that TCM nursing significantly reduced SDS scores (MD = −5.94, 95%CI −6.96 to −4.92, *p* < 0.00001) (Figure 4B), full sample. To explore the source of heterogeneity, we performed outlier detection via leave-one-out sensitivity analysis. After excluding the outlier study by Dong and Guan (32), the heterogeneity was reduced to an acceptable level (*p* = 0.13, *I*^2^ = 36%). The fixed-effect model for this sensitivity analysis showed that the SDS score of the experimental group was significantly lower than that of the control group (MD = −5.74, 95%CI −6.39 to −5.09, *p* < 0.00001). The pooled effect size remained stable, indicating that the results were robust (Figure 4).

##### 3.4.2.2 Quality of life (KPS score)

**Figure 5:**
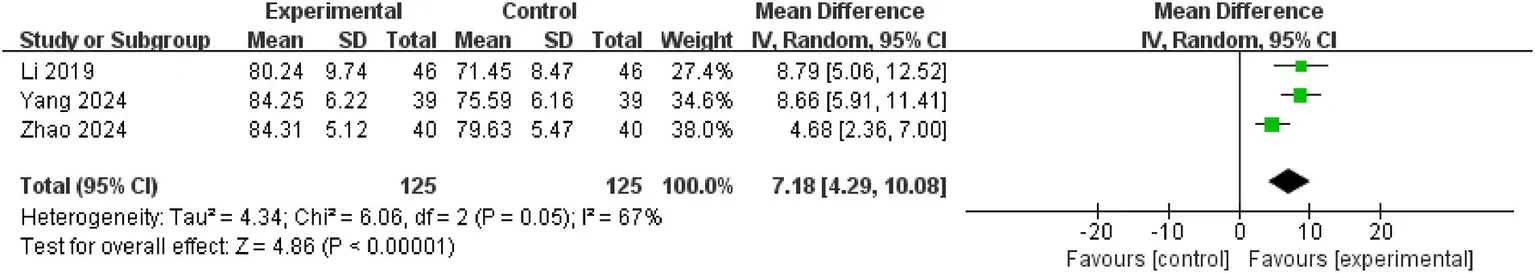
Forest plot of Karnofsky Performance Status (KPS) scores (primary analysis including all three studies, random-effects model).

**Figure 6:**
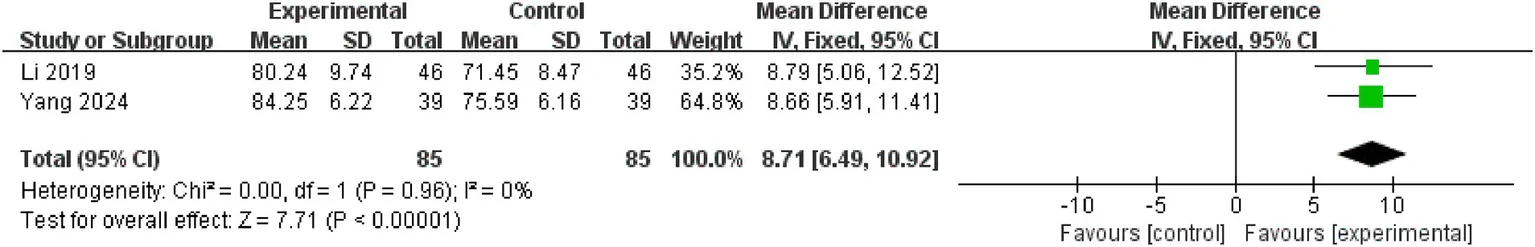
Sensitivity analysis for KPS scores (after excluding Zhao et al.).

Three studies (21, 23, 28) reported KPS scores for evaluating patients’ quality of life. The full-sample primary analysis showed high heterogeneity (*p* = 0.05, *I*^2^ = 67%), and the random-effects model showed that TCM nursing significantly improved KPS scores (MD = 7.18, 95%CI 4.29–10.08, *p* < 0.00001) (Figure 5). Leave-one-out sensitivity analysis identified Zhao et al. (21) as the outlier study. After excluding this study, heterogeneity was completely eliminated (*p* = 0.96, *I*^2^ = 0%). The fixed-effect model for this sensitivity analysis showed that the KPS score of the experimental group was significantly higher than that of the control group (MD = 8.71, 95%CI 6.49–10.92, *p* < 0.00001) (Figure 6). The direction and statistical significance of the effect remained consistent, confirming the robustness of the result.

##### 3.4.2.3 Pain (VAS score)

**Figure 7:**
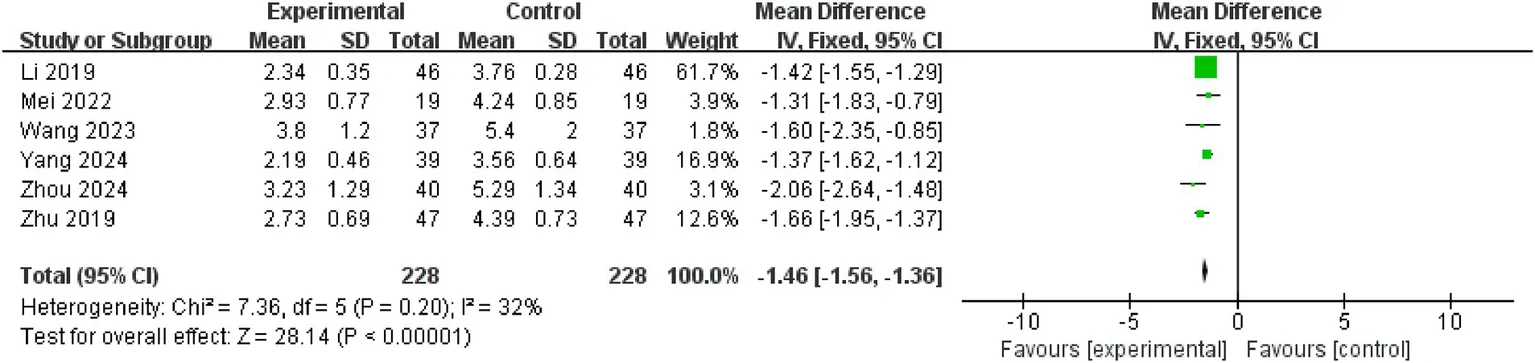
Forest plot of visual analog scale (VAS) pain scores.

Six studies (19, 20, 23, 25, 27, 28) reported VAS scores to evaluate patients’ pain degree. The heterogeneity test showed low heterogeneity among studies (*p* = 0.20, *I*^2^ = 32%), and the fixed-effect model was used for analysis. The results showed that the VAS score of the experimental group was significantly lower than that of the control group (MD = −1.46, 95%CI −1.56 to −1.36, *p* < 0.00001) (Figure 7).

##### 3.4.2.4 Incidence of gastrointestinal reactions

**Figure 8:**
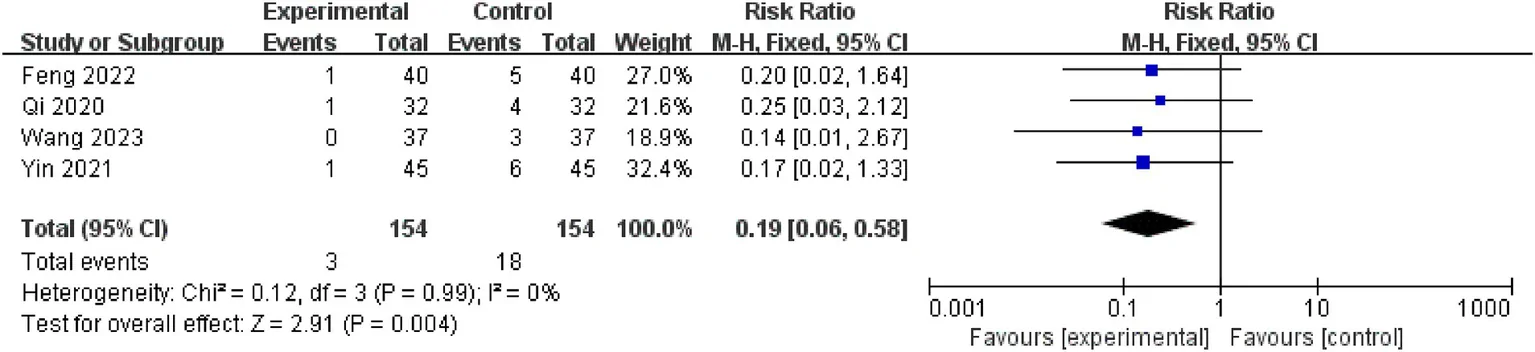
Forest plot of gastrointestinal reactions.

Four studies (22, 25, 26, 31) reported the incidence of gastrointestinal reactions in patients. The heterogeneity test showed no heterogeneity between studies (*p* = 0.99, *I*^2^ = 0%), and the fixed-effect model was used for analysis. The results showed that the incidence of gastrointestinal reactions in the experimental group was significantly lower than in the control group (RR = 0.19, 95%CI 0.06–0.58, *p* = 0.004) (Figure 8).

#### 3.4.3 Structural quality

Two studies (22, 32) reported implementation of standardized Traditional Chinese Medicine nursing training systems for nursing staff, including professional knowledge and standardized operating procedures. Eight studies (22, 23, 25–28, 30, 33) reported standardized management of the inpatient environment including temperature, humidity, and ventilation aimed at improving respiratory comfort. However, reporting on structure-related elements was generally poor. No studies reported on the availability of Traditional Chinese Medicine equipment or supplies, staffing levels, or qualification requirements—factors that the Donabedian model identifies as foundational to quality care. This underreporting limits our ability to assess how structural factors may influence outcomes in Traditional Chinese Medicine nursing.

### 3.5 Subgroup analysis

**Figure 9:**
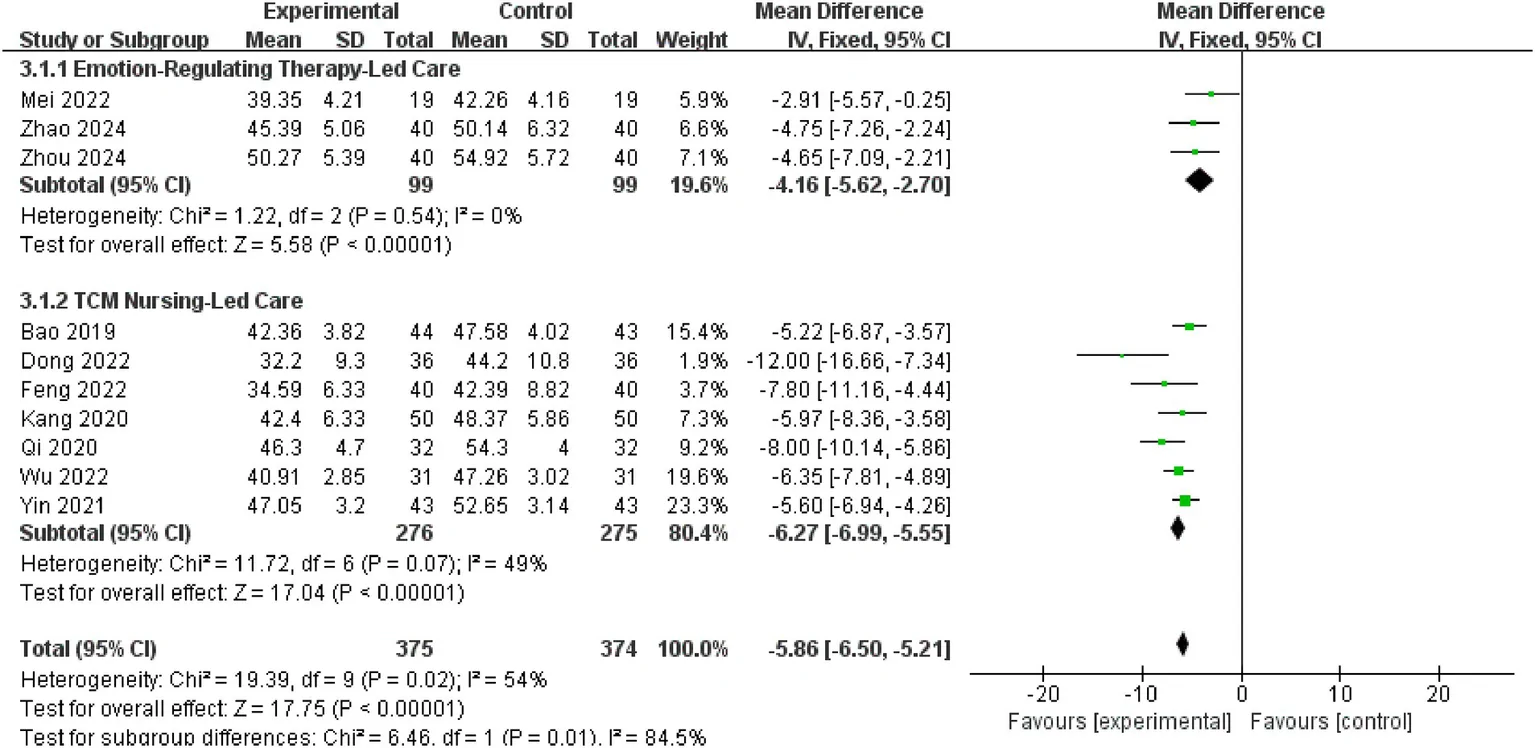
Subgroup analysis for Self-Rating Depression Scale (SDS) scores by intervention type.

Subgroup analysis was conducted for SDS score (the main outcome with heterogeneous sources) according to the intervention type, including three studies (20, 21, 27) of emotion regulation intervention (five-element music, emotional nursing) and seven studies (22, 24, 26, 29, 31–33) of TCM nursing-led comprehensive care (acupoint application, syndrome differentiation nursing, integrated Chinese and Western medicine nursing). The results showed that both intervention types could significantly reduce the patients’ SDS scores (corrected *p* < 0.0083). The emotion-regulation intervention subgroup showed a moderate improvement (MD = −4.16, 95%CI −5.62 to −2.70, *I*^2^ = 0%), while the TCM comprehensive care subgroup showed a more significant improvement (MD = −6.27, 95%CI −6.99 to −5.55, *I*^2^ = 49%). The difference between the two subgroups was statistically significant (*p* = 0.01), indicating that comprehensive TCM care produced significantly larger antidepressant effects than emotion regulation alone (Figure 9).

### 3.6 Sensitivity analysis

Sensitivity analysis was performed using two methods: sequential omission of a single study and exclusion of high-risk bias studies. For the SDS score, after omitting Dong and Guan’s study (32), the heterogeneity was significantly reduced, and the pooled effect size remained stable, confirming this study as the main source of heterogeneity. For the KPS score, after omitting Zhao et al.’s study (21), the heterogeneity was completely eliminated, and the pooled effect size remained significant (*I*^2^ = 0%, *p* < 0.05), confirming this study as the main source of heterogeneity. Sensitivity analysis excluding five high-risk bias studies (19, 24–26, 31) showed that the pooled effect sizes of all outcome indicators remained statistically significant (*p* < 0.05) with no significant change in effect size direction. Sensitivity analysis of other outcome measures (SAS score, VAS score, nursing compliance, incidence of gastrointestinal reactions) showed that the pooled results were not significantly affected by a single study or high-risk bias studies, indicating that the meta-analysis results were robust and reliable (Figure 6).

### 3.7 Publication bias

**Figure 10:**
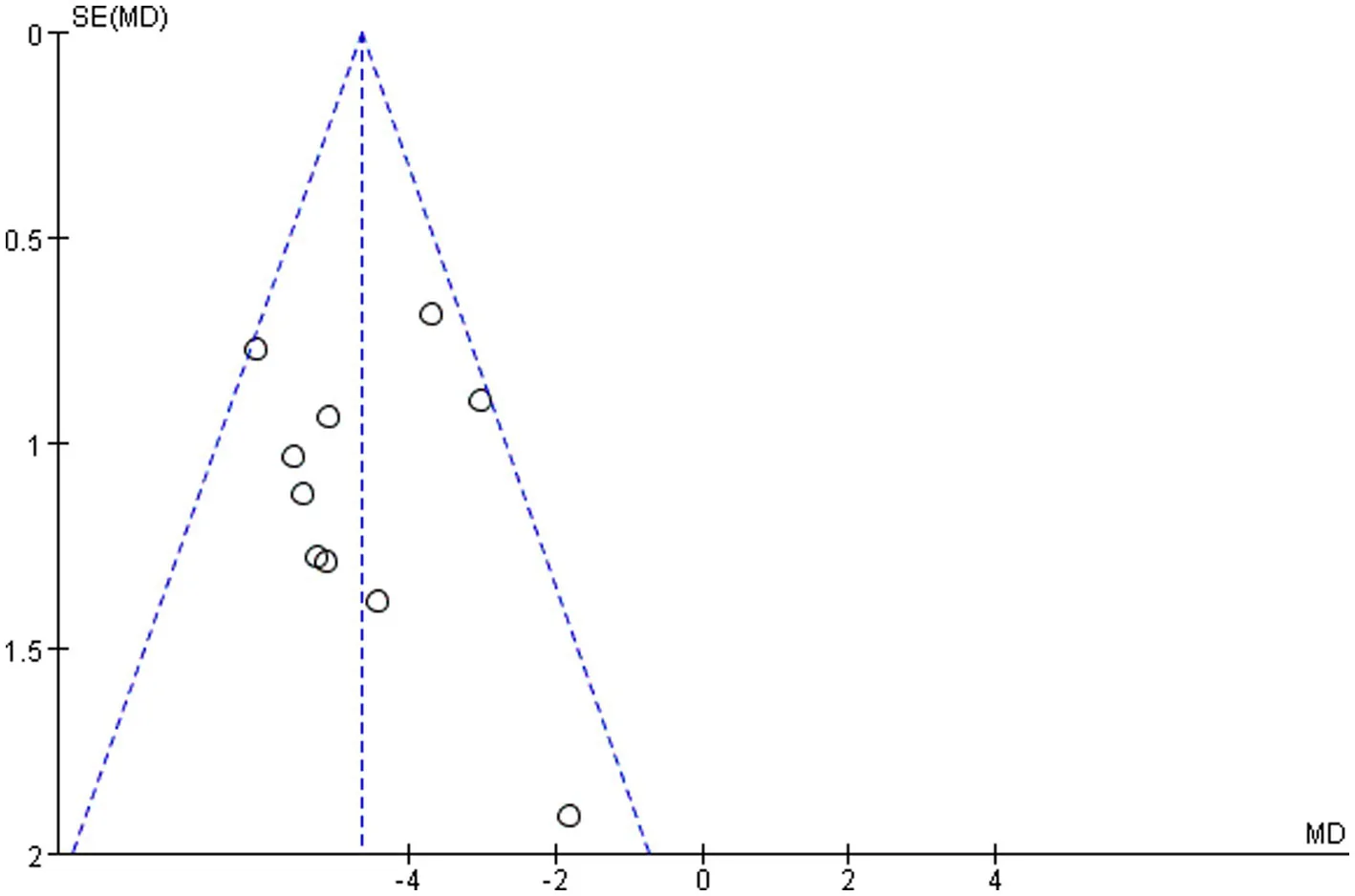
Funnel plot for SAS scores.

Ten studies (20–22, 24, 26, 27, 29, 31–33) with SAS score as the outcome measure were selected for publication bias evaluation. The funnel plot was basically symmetric, and Egger’s test (*p* = 0.074) and Begg’s test (*p* = 0.107) both showed a *p*-value of >0.05, indicating that there was no significant publication bias in the included studies (Figure 10).

## 4 Discussion

### 4.1 Efficacy of TCM nursing for lung cancer and multi-dimensional analysis based on the Donabedian model

This systematic review and meta-analysis, using the Donabedian Structure-Process-Outcome model as an analytical lens, provides two main contributions to understanding TCM nursing for lung cancer. First, it confirms the overall efficacy of TCM nursing across multiple patient-reported outcomes. Second, and more importantly, the application of the Donabedian model reveals that certain process factors—particularly intervention type (verified by subgroup analysis) and, potentially, intervention timing (suggested by sensitivity analysis)—may contribute to the heterogeneity observed in the evidence base, a finding that traditional meta-analytic approaches alone could not have systematically identified.

Consistent with previous reviews (34–36), our findings demonstrate that in terms of outcome quality, TCM nursing can significantly improve the quality of life of lung cancer patients (increase KPS score), alleviate negative emotions (reduce SAS and SDS scores), relieve cancer-related pain (reduce VAS score), and reduce the incidence of chemotherapy-induced gastrointestinal reactions. These findings align with the theoretical basis of TCM nursing, which emphasizes holistic care and syndrome differentiation—tailoring interventions to patients’ specific constitutional patterns and symptom profiles (8, 9). The consistency of positive effects across diverse outcomes suggests that TCM nursing addresses the multidimensional nature of the cancer experience, rather than targeting isolated symptoms. However, the magnitude of these effects must be interpreted in light of the substantial heterogeneity observed, and the Donabedian framework enabled us to systematically explore its sources rather than viewing heterogeneity as merely a statistical limitation (37, 38). In terms of process quality, subgroup analysis confirmed that intervention type (comprehensive TCM care vs. emotion-regulation alone) significantly moderated treatment outcomes (*p* = 0.01), while sensitivity analysis suggested that intervention timing may also play a role. Regarding intervention type, comprehensive TCM care combining multiple modalities such as acupoint application, herbal interventions, and emotional support showed significantly larger effects on depression than emotion-regulation alone, suggesting that the synergistic effects of multimodal TCM interventions may be particularly important for addressing the complex psychological challenges faced by lung cancer patients. Depression in cancer patients (39) is multifactorial, arising from disease-related symptoms, treatment side effects, existential concerns, and social disruption (40), and comprehensive TCM nursing may more effectively target these multiple pathways than single-modality approaches by simultaneously addressing physical symptoms through acupoint application (41), emotional distress through emotional nursing, and constitutional imbalance through syndrome differentiation. Notably, the sensitivity analysis identified Dong and Guan (32) as an outlier; however, the specific reasons for this divergence could not be determined due to incomplete reporting of intervention characteristics in the original studies. In terms of structural quality, and in contrast to the relatively rich data on outcomes and some process factors, structural quality indicators were poorly reported across included two studies (22, 32) describing standardized TCM nursing training for staff and eight studies mentioning environmental management (22, 23, 25–28, 30, 33), while no studies reported on the availability of TCM equipment or supplies, staffing levels, or qualification requirements—factors that the Donabedian model identifies as foundational to quality care. This underreporting is problematic because structure enables process, and without adequately trained staff and appropriate facilities, even the best-designed interventions cannot be delivered effectively (42, 43). The lack of structural data in the current evidence base limits our understanding of the contextual factors that may influence TCM nursing outcomes, leaving questions unanswered about whether practitioner training levels affect treatment effects or whether dedicated TCM nursing facilities enhance patient engagement and adherence.

### 4.2 What the Donabedian model reveals about heterogeneity in TCM nursing evidence

Beyond verifying the efficacy of TCM nursing, the Donabedian model further provides a systematic perspective for exploring the heterogeneity of existing evidence, which is the core contribution of this study. Applying the Donabedian model to this meta-analysis yielded insights that extend beyond traditional heterogeneity exploration. First, by categorizing data into structure, process, and outcome dimensions, the model enabled a structured approach to identifying which specific factors contribute to heterogeneity. This analysis revealed that process factors—particularly intervention type (verified by a formal subgroup analysis) and intervention timing (as indicated by a sensitivity analysis)—may partially contribute to the observed variation in treatment effects. Second, the model highlighted critical gaps in the current evidence base: while outcome data are relatively rich, structural factors such as staff training, equipment availability, and qualification requirements are severely underreported. This finding suggests that future research cannot fully explain variations in TCM nursing efficacy without systematically collecting and reporting structural data. Third, the compatibility between the Donabedian model’s three dimensions and TCM nursing’s holistic philosophy (environment-disease-human) suggests that this model is particularly well-suited for organizing future inquiries into how TCM nursing works, for whom, and under what conditions.

### 4.3 Limitations of the study

This study has several limitations that should be considered when interpreting the findings. First, the methodological quality of the included RCTs is generally low, with all studies not reporting allocation concealment and some studies having a high-risk bias due to no blinding. Although sensitivity analyses showed the robustness of the results, this may still affect the credibility of the evidence. Second, the reporting of structure-related elements in the included studies is seriously insufficient, and quantitative analysis of the influence of structural factors on TCM nursing efficacy cannot be conducted, which limits the in-depth application of the Donabedian model. Third, all included studies were conducted in China, and the research results are generally not generalizable to other medical systems and cultural backgrounds where TCM nursing is less prevalent. Fourth, the number of included studies for some outcomes is small: only three studies were included for KPS score and nursing compliance, and the emotion-regulation subgroup in the SDS subgroup analysis also included only three studies. A small evidence base may reduce the stability, precision, and generalizability of the pooled estimates, so the results of these outcomes should be interpreted with caution. More high-quality RCTs focus on these outcomes are needed to generate more precise and definitive evidence. Fifth, although subgroup analysis was conducted according to intervention type, the potential influence of other process factors such as intervention frequency, intervention dose, and disease stage on heterogeneity was not explored, and residual unexplained heterogeneity may remain. In particular, the inference about intervention timing is only derived from single-study sensitivity analysis and lacks verification by formal subgroup analysis or meta-regression. Sixth, this study is a retrospective secondary analysis of published data, and prospective studies designed according to the Donabedian model are needed to further verify the research conclusions.

## 5 Conclusion

This systematic review and meta-analysis provide evidence that TCM nursing significantly improves quality of life, psychological wellbeing, pain, and gastrointestinal symptoms in lung cancer patients, although these overall effects mask substantial heterogeneity that has important implications for both research and practice. Applying the Donabedian Structure-Process-Outcome model as an analytical framework revealed that process factors, particularly intervention type (formally verified by subgroup analysis), are important moderators of treatment effects and partially explain the observed heterogeneity. Sensitivity analysis indicates that intervention timing is a potential source of heterogeneity, which requires further verification. In contrast, structural factors are underreported in the current evidence base, limiting our understanding of the contextual conditions that enable effective TCM nursing. These findings suggest that future research should move beyond simply asking whether TCM nursing works, to investigating how, for whom, and under what conditions it works best, and the Donabedian model provides a useful framework for organizing this inquiry. By systematically attending to structure, process, and outcome dimensions, researchers can build a more robust evidence base that supports both the scientific understanding and clinical application of TCM nursing for lung cancer patients.

## Data Availability

The original contributions presented in the study are included in the article/Supplementary material, further inquiries can be directed to the corresponding author/s.
