# Supplementary material for: Efficacy of traditional Chinese medicine nursing for lung cancer: a systematic review and meta-analysis using the Donabedian model to investigate heterogeneity
Source: Front Public Health. 2026 Aug 26;14:1833561. doi: 10.3389/fpubh.2026.1833561 (PMC13553233; doi:10.3389/fpubh.2026.1833561)
Supplement: Supplementary file 1 [file Table_1.DOC]

## **Appendix 1. Detailed Search Strategies for Each Database**

**PubMed**

**Broad search (for initial identification):**

**("Lung Neoplasms"[MeSH] OR "lung cancer"[Title/Abstract] OR "pulmonary cancer"[Title/Abstract] OR "non-small cell lung cancer"[Title/Abstract] OR "NSCLC"[Title/Abstract]) AND ("Medicine, Chinese Traditional"[MeSH] OR "traditional Chinese medicine nursing"[Title/Abstract] OR "TCM nursing"[Title/Abstract] OR "syndrome differentiation nursing"[Title/Abstract] OR "acupoint application"[Title/Abstract] OR "five-element music"[Title/Abstract] OR "emotional nursing"[Title/Abstract])**

**Restricted search (for full-text screening):**

**("Lung Neoplasms"[MeSH] OR "lung cancer"[Title/Abstract] OR "pulmonary cancer"[Title/Abstract] OR "non-small cell lung cancer"[Title/Abstract] OR "NSCLC"[Title/Abstract]) AND ("Medicine, Chinese Traditional"[MeSH] OR "traditional Chinese medicine nursing"[Title/Abstract] OR "TCM nursing"[Title/Abstract] OR "syndrome differentiation nursing"[Title/Abstract] OR "acupoint application"[Title/Abstract] OR "five-element music"[Title/Abstract] OR "emotional nursing"[Title/Abstract]) AND ("Quality of Life"[MeSH] OR "Karnofsky Performance Status"[Title/Abstract] OR "KPS"[Title/Abstract] OR "Anxiety"[MeSH] OR "SAS"[Title/Abstract] OR "Depression"[MeSH] OR "SDS"[Title/Abstract] OR "Pain"[MeSH] OR "VAS"[Title/Abstract] OR "gastrointestinal reactions"[Title/Abstract] OR "nursing compliance"[Title/Abstract]) AND ("Randomized Controlled Trial"[Publication Type] OR "RCT"[Title/Abstract] OR "randomized"[Title/Abstract] OR "controlled clinical trial"[Publication Type])**

**Web of Science**

**Broad search (for initial identification):**

TS=("lung cancer" OR "pulmonary cancer" OR "NSCLC" OR "non-small cell lung cancer") AND TS=("traditional Chinese medicine nursing" OR "TCM nursing" OR "syndrome differentiation nursing" OR "acupoint application" OR "five-element music" OR "emotional nursing")

**Restricted search (for full-text screening):**

**TS=("lung cancer" OR "pulmonary cancer" OR "NSCLC" OR "non-small cell lung cancer") AND TS=("traditional Chinese medicine nursing" OR "TCM nursing" OR "syndrome differentiation nursing" OR "acupoint application" OR "five-element music" OR "emotional nursing") AND TS=("quality of life" OR "KPS" OR "anxiety" OR "SAS" OR "depression" OR "SDS" OR "pain" OR "VAS" OR "gastrointestinal reactions" OR "nursing compliance") AND TS=("randomized controlled trial" OR "RCT" OR "randomized")**

**Embase**

**Broad search (for initial identification):**

('lung cancer'/exp OR 'lung tumor' OR 'NSCLC') AND ('traditional chinese medicine'/exp OR 'TCM nursing' OR 'acupoint application' OR 'five-element music')

**Restricted search (for full-text screening):**

**('lung cancer'/exp OR 'lung tumor' OR 'NSCLC') AND ('traditional chinese medicine'/exp OR 'TCM nursing' OR 'acupoint application' OR 'five-element music') AND ('quality of life'/exp OR KPS OR anxiety OR SAS OR depression OR SDS OR pain OR VAS) AND ('randomized controlled trial'/exp OR RCT)**

**Cochrane Library**

**Broad search (for initial identification):**

(lung cancer OR pulmonary cancer OR NSCLC) AND (TCM nursing OR syndrome differentiation nursing OR acupoint application OR five-element music OR emotional nursing)

**Restricted search (for full-text screening):**

**(lung cancer OR pulmonary cancer OR NSCLC) AND (TCM nursing OR syndrome differentiation nursing OR acupoint application OR five-element music OR emotional nursing) AND (KPS OR SAS OR SDS OR VAS OR gastrointestinal reactions) AND (RCT OR randomized)**

**CNKI（中国知网）**

**Broad search (for initial identification):**

(主题='肺癌'+'肺恶性肿瘤'+'非小细胞肺癌'+'NSCLC')*(主题='中医护理'+'辨证护理'+'穴位贴敷'+'五行音乐'+'情志护理'+'中西医结合护理')

**Restricted search (for full-text screening):**

**(主题='肺癌'+'肺恶性肿瘤'+'非小细胞肺癌'+'NSCLC')*(主题='中医护理'+'辨证护理'+'穴位贴敷'+'五行音乐'+'情志护理'+'中西医结合护理')*(主题='生活质量'+'KPS'+'焦虑'+'SAS'+'抑郁'+'SDS'+'疼痛'+'VAS'+'胃肠道反应'+'依从性')*(主题='随机对照试验'+'RCT'+'随机')**

**VIP（维普）**

**Broad search (for initial identification):**

M=(肺癌 + 肺恶性肿瘤 + 非小细胞肺癌 + NSCLC) * M=(中医护理 + 辨证护理 + 穴位贴敷 + 五行音乐 + 情志护理 + 中西医结合护理)

**Restricted search (for full-text screening):**

**M=(肺癌 + 肺恶性肿瘤 + 非小细胞肺癌 + NSCLC) * M=(中医护理 + 辨证护理 + 穴位贴敷 + 五行音乐 + 情志护理 + 中西医结合护理) * M=(生活质量 + KPS + 焦虑 + SAS + 抑郁 + SDS + 疼痛 + VAS + 胃肠道反应 + 依从性) * M=(随机对照试验 + RCT + 随机)**

**WanFang（万方）**

**Broad search (for initial identification):**

主题:("肺癌" OR "肺恶性肿瘤" OR "非小细胞肺癌" OR "NSCLC") AND 主题:("中医护理" OR "辨证护理" OR "穴位贴敷" OR "五行音乐" OR "情志护理" OR "中西医结合护理")

**Restricted search (for full-text screening):**

**主题:("肺癌" OR "肺恶性肿瘤" OR "非小细胞肺癌" OR "NSCLC") AND 主题:("中医护理" OR "辨证护理" OR "穴位贴敷" OR "五行音乐" OR "情志护理" OR "中西医结合护理") AND 主题:("生活质量" OR "KPS" OR "焦虑" OR "SAS" OR "抑郁" OR "SDS" OR "疼痛" OR "VAS" OR "胃肠道反应" OR "依从性") AND 主题:("随机对照试验" OR "RCT" OR "随机")**
